# An Informational Theoretical Approach to the Entropy of Liquids and Solutions

**DOI:** 10.3390/e20070514

**Published:** 2018-07-09

**Authors:** Arieh Ben-Naim

**Affiliations:** Department of Physical Chemistry, The Hebrew University of Jerusalem, Edmond J. Safra Campus, Givat Ram, Jerusalem 9190401, Israel; ariehbennaim@gmail.com

**Keywords:** entropy, information theory, liquids, solutions

## Abstract

It is well known that the statistical mechanical theory of liquids has been lagging far behind the theory of either gases or solids, See for examples: Ben-Naim (2006), Fisher (1964), Guggenheim (1952) Hansen and McDonald (1976), Hill (1956), Temperley, Rowlinson and Rushbrooke (1968), O’Connell (1971). Information theory was recently used to derive and interpret the entropy of an ideal gas of simple particles (i.e., non-interacting and structure-less particles). Starting with Shannon’s measure of information (SMI), one can derive the *entropy* function of an ideal gas, the same function as derived by Sackur (1911) and Tetrode (1912). The new deviation of the same entropy function, based on SMI, has several advantages, as listed in Ben-Naim (2008, 2017). Here we mention two: First, it provides a simple interpretation of the various terms in this entropy function. Second, and more important for our purpose, this derivation may be extended to any system of interacting particles including liquids and solutions. The main idea is that once one adds intermolecular interactions between the particles, one also adds correlations between the particles. These correlations may be cast in terms of mutual information (MI). Hence, we can start with the informational theoretical interpretation of the entropy of an ideal gas. Then, we add correction due to correlations in the form of MI between the locations of the particles. This process preserves the interpretation of the entropy of liquids and solutions in terms of a measure of information (or as an average uncertainty about the locations of the particles). It is well known that the entropy of liquids, any liquids for that matter, is lower than the entropy of a gas. Traditionally, this fact is interpreted in terms of order-disorder. The lower entropy of the liquid is interpreted in terms of higher degree of order compared with that of the gas. However, unlike the transition from a solid to either a liquid, or to a gaseous phase where the order-disorder interpretation works well, the same interpretation would not work for the liquid-gas transition. It is hard, if not impossible, to argue that the liquid phase is more “ordered” than the gaseous phase. In this article, we interpret the lower entropy of liquids in terms of SMI. One outstanding liquid known to be a structured liquid, is water, according to Ben-Naim (2009, 2011). In addition, heavy water, as well as aqueous solutions of simple solutes such as argon or methane, will be discussed in this article.

## 1. Introduction

**Figure 1:**
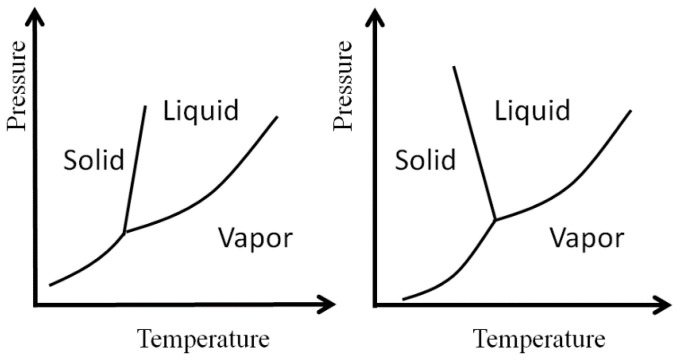
Schematic phase diagram of a normal liquid (**Left**) and water (**Right**). Note the different slopes of the solid-liquid equilibrium lines.

It is well known that the entropy of liquids is lower than the entropy of the corresponding vapor of the same substances at a given temperature and pressure [1,2,3,4,5,6,7,8,9,10]. This fact is manifested in the slope of the liquid (*l*)–gas (*g*) coexistence curve in the phase diagram. The slope of the *P(T)* curve, is given by the Clapeyron equation:

(1) $$\;{\left(\frac{dP}{dT}\right)}_{eq}=\frac{\Delta S_{v}}{\Delta V_{v}}>0$$

where $\Delta S_{v}$ is the entropy of vaporization, $\Delta V_{v}$ is the volume of vaporization, $\Delta V_{v}=V\left(g\right)-V\left(l\right)$, and the derivative is taken along the liquid-gas coexistence curve in the phase diagram, Figure 1.

Since $\Delta V_{v}$ is always very large and positive, the positive slope of the liquid-gas equilibrium curve means that $\Delta S_{v}$ is always positive. It should be noted that Equation (1) is an exact equation. An approximate equation, known as the Clausius-Clapeyron equation, may be obtained by assuming that $V_{g}\gg V_{l}$, and that the vapor is an ideal gas, i.e., $V_{g}=RT/P$, where *R* is the gas constant, and *P* the pressure. Using this approximation, one gets:

(2) $$\;{\left(\frac{dP}{dT}\right)}_{eq}=\frac{P\Delta S_{v}}{RT}=\frac{P\Delta H_{v}}{RT^{2}}$$

where we used the equality $T\Delta S_{v}=\Delta H_{v}$.

Another well-known approximate empirical law is the Trouton Law. It states that the entropy of vaporization at one atmospheric pressure of many liquids is almost constant:

(3) $$\Delta S_{v}\approx 85-87/J\;{\mathrm{mol}}^{-1}{\;K}^{-1}$$

Table 1 shows a few values of the entropy of vaporization of some liquids. Note that the values of $\Delta S_{v\;}$ for water, ethanol, and methanol are much larger than the values for the other liquids.

Traditionally, the positive value of the entropy of vaporization and the entropy of sublimation is interpreted in terms of order-disorder. Following the erroneous, but very common interpretation of entropy as a measure of disorder, one concludes that the liquid state is more “ordered” than the gaseous phase.

Unfortunately, unlike the case of sublimation (the transition from solid to gas), where the order-disorder argument seems to work, in the case of vaporization, the order-disorder argument fails. It is hard, if not impossible, to argue that the liquid phase is more ordered than the gaseous phase.

In this article, we propose a novel interpretation of the positive entropy of vaporization in terms of *mutual information*, as per Shannon [11] and Ben-Naim [12]. It will be shown that in general, the transition from a state of non-interaction between particles to a state when intermolecular interaction is operative, the Shannon measure of information (SMI) always decreases. This argument is also carried over from SMI into the entropy, the latter being viewed as a special case of the former.

In the next section, we shall briefly outline the derivation of entropy from the SMI. We shall then show in Section 3, that whenever we “turn on” the interactions among the particles, the entropy of the system will always decrease. A special case of non-ideal gas will be discussed in Section 4.

## 2. Entropy of an Ideal Gas of Simple Particles

In this section, we outline the procedure by which we obtain the entropy of an ideal gas from the SMI. We discuss here simple particles, i.e., particles that have no internal degrees of freedom. This means that the particles are spherical and that the full specification of the (classical) microstate of a system of *N* particles required 3*N* coordinates of locations and 3*N* velocities (or momenta).

The procedure of obtaining the entropy of an ideal gas from SMI consists of essentially four steps:

1. Calculation of the locational SMI at equilibrium
2. Calculation of the momentum SMI at equilibrium
3. Adding a correction due to the uncertainty principle
4. Adding a correction due to the indistinguishability of the particles

The resulting SMI of both locations and momenta at equilibrium leads to the entropy function, S(E,V,N), or S(T,V,N) which was originally derived from the Boltzmann entropy by Sackur [13], and Tetrode [14].

We shall be very brief here; a more detailed derivation is available in Ben-Naim [12,15].

### 2.1. The Locational SMI of a Particle in a 1D Box of Length L

Suppose we have a particle confined to a one-dimensional (1D) “box” of length *L*. We can define the continuous SMI by:

(4)  H(X)=−∫f(x)logf(x)dx

where f(x)dx is the probability of finding the particle between *x* and *x + dx.* Next, we calculate the density distribution, which maximizes the locational SMI, H(X) in (4). The result is:

(5) $$\;f_{eq}\left(x\right)=\frac{1}{L}$$

We identify the distribution that maximizes the SMI as the equilibrium (*eq*) distribution by substituting (5) in (4):

(6)  H(locations in 1D)=logL

We now acknowledge that the location *X* of the particle cannot be determined with absolute accuracy, i.e., there exists a small interval $h_{x}$ within which we do not care where the particle is. Therefore, we correct Equation (6) by subtracting $\log h_{x}$. Thus, we write instead of (6),

(7) $$\;H\left(X\right)=\log L-\log h_{x}$$

**Figure 2:**
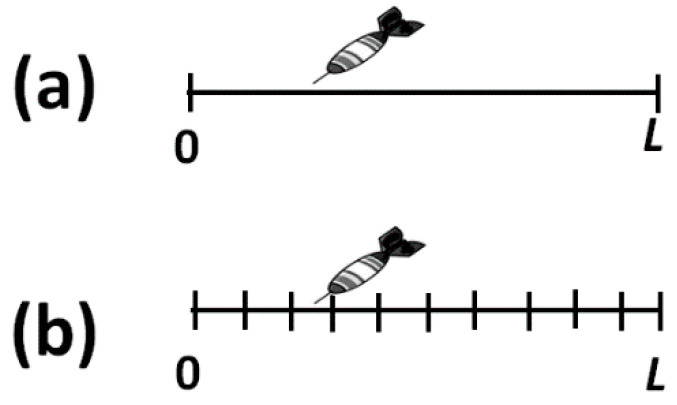
Transition from the continuous to the discrete case. (**a**) A dart hits a one-dimensional segment of length *L*. There are infinite possible locations for the dart; (**b**) Passage from the infinite to the discrete description of the states.

In Equation (7) we effectively defined H(X) for the finite number of intervals n=L/h. See Figure 2. Note that when $h_{x}\to 0,\;H\left(X\right)$ diverges to infinity. Here, we do not take the mathematical limit, but we stop at $h_{x}$ small enough, but not zero. Note also that in writing Equation (7) we do not have to specify the units of length, as long as we use the same units for *L* and $h_{x}$.

### 2.2. The Velocity SMI of a Particle in a 1D “Box” of Length L

Next, we calculate the probability distribution that maximizes the continuous SMI, subject to two conditions:

(8) $$\;\overset{\infty }{\underset{-\infty }{\int }}f\left(x\right)dx=1$$

(9) $$\;\overset{\infty }{\underset{-\infty }{\int }}x^{2}f\left(x\right)dx=\sigma^{2}=constant$$

The result is the normal distribution:

(10) $$\;f_{eq}\left(x\right)=\frac{\exp\left[-x^{2}/2\sigma^{2}\right]}{\sqrt{2\pi \sigma^{2}}}$$

Again, we use the subscript *eq* for equilibrium. Applying this result to a classical particle having average kinetic energy $\frac{m<v_{x}^{2}>}{2}$, and using the relationship between the standard deviation $\sigma^{2}$ and the temperature of the system:

(11) $$\sigma^{2}=\frac{k_{B}T}{m},$$

we get the equilibrium velocity distribution of one particle in a 1D system:

(12) $$\;f_{eq}\left(v_{x}\right)=\sqrt{\frac{m}{2\pi k_{B}T}}\;\exp\left[\frac{-mv_{x}^{2}}{2k_{B}T}\right]$$

Here, $k_{B}$ is the Boltzmann constant, *m* is the mass of the particle, and *T* the absolute temperature. The value of the continuous SMI for this probability density is:

(13) $$\;H_{max}\left(\mathrm{velocity}\;\mathrm{in}\;1D\right)=\frac{1}{2}\log\left(2\pi ek_{B}T/m\right)$$

Similarly, we can write the momentum distribution in 1D, by transforming from $v_{x}\to p_{x}=mv_{x}$, to get:

(14) $$\;f_{eq}\left(p_{x}\right)=\frac{1}{\sqrt{2\pi mk_{B}T}}\;\exp\left[\frac{-p_{x}^{2}}{2mk_{B}T}\right]$$

and the corresponding maximum SMI:

(15) $$\;H_{max}\left(\mathrm{momentum}\;\mathrm{in}\;1D\right)=\frac{1}{2}\log\left(2\pi emk_{B}T\right)$$

Again, recognizing the fact that there is a limit to the accuracy within which we can determine the velocity (or the momentum) of the particle, we correct the expression in (15) by subtracting log $h_{p},\;$ where $h_{p}$ is a small, but finite interval:

(16) $$\;H_{max}\left(\mathrm{momentum}\;\mathrm{in}\;1D\right)=\frac{1}{2}\log\left(2\pi emk_{B}T\right)-\log h_{p}$$

Note again that if we choose the units of $h_{p}$ of momentum as mass length/time, the same as of $\sqrt{mk_{B}T}$, then the whole expression under the logarithm will be a pure number.

### 2.3. Combining the SMI for the Location and Momentum of a Particle in a 1D System

We now combine the two results. Assuming that the location and the momentum (or velocity) of the particles are independent events, we write:

(17) $$H_{max}\left(\mathrm{location}\;\mathrm{and}\;\mathrm{momentum}\right)=H_{max}\left(\mathrm{location}\right)\;+\;H_{max}\left(\mathrm{momentum}\right)=\;\log\left[\frac{L\sqrt{2\pi emk_{B}T}}{h_{x}h_{p}}\right]$$

Recall that $h_{x}$ and $h_{p}$ were chosen to eliminate the divergence of the SMI when the location and momentum were treated as continuous random variables.

In writing (17), we assume that the location and the momentum of the particle are independent. However, quantum mechanics imposes restriction on the accuracy in determining both the location *x* and the corresponding momentum $p_{x}$. In Equations (7) and (16), $h_{x}$ and $h_{p}$ were introduced because we did not care to determine the location and the momentum with an accuracy better than $h_{x}$ and $h_{p}$, respectively. Now, we must acknowledge that nature imposes upon us a limit on the accuracy with which we can determine simultaneously the location and the corresponding momentum. Thus, in Equation (17), $h_{x}$ and $h_{p}$ cannot both be arbitrarily small, but their product must be of the order of Planck constant $h=6.626\;\times \;10^{-34}\;J\;s$. Thus, we set:

(18) $$\;h_{x}h_{p}\approx h$$

And instead of (17), we write:

(19) $$\;H_{max}\left(\mathrm{location}\;\mathrm{and}\;\mathrm{momentum}\right)=\log\left[\frac{L\sqrt{2\pi emk_{B}T}}{h}\right]$$

### 2.4. The SMI of a Particle in a Box of Volume V

We consider again one simple particle in a cubic box of volume *V*. We assume that the location of the particle along the three axes *x*, *y* and *z* are independent. Therefore, we can write the SMI of the location of the particle in a cube of edges *L*, and volume *V* as:

(20) $$\;H\left(\mathrm{location}\;\mathrm{in}\;3D\right)=3H_{max}\left(\mathrm{location}\;\mathrm{in}\;1D\right)$$

Similarly, for the momentum of the particle, we assume that the momentum (or the velocity) along the three axes *x*, *y* and *z* are independent. Hence, we write:

(21) $$\;H_{max}\left(\mathrm{momentum}\;\mathrm{in}\;3D\right)=3H_{max}\left(\mathrm{momentum}\;\mathrm{in}\;1D\right)$$

We combine the SMI of the locations and momenta of one particle in a box of volume *V*, taking into account the uncertainty principle. The result is:

(22) $$\;H_{max}\left(\mathrm{location}\;\mathrm{and}\;\mathrm{momentum}\;\mathrm{in}\;3D\right)=3\log\left[\frac{L\sqrt{2\pi emk_{B}T}}{h}\right]$$

### 2.5. The SMI of Locations and Momenta of N Independent Particles in a Box of Volume V

The next step is to proceed from one particle in a box to *N* independent particles in a box of volume *V*. Giving the location (x,y,z), and the momentum $\left(p_{x},p_{y},p_{z}\right)$ of one particle within the box, we say that we know the microstate of the particle. If there are *N* particles in the box, and if their microstates are independent, we can write the SMI of *N* such particles simply as *N* times the SMI of one particle, i.e.,

(23)  SMI( N independent particles)=N×SMI(one particle)

This equation would have been correct if the microstates of all the particles were independent. In reality, there are always correlations between the microstates of all the particles; one is due to the indistinguishability of the particles, the second is due to intermolecular interactions among all the particles. We shall discuss these two sources of correlation separately. In this section, we introduce the correlation due to indistinguishability. In the next section, we introduce the correlation due to intermolecular interactions.

Recall that the microstate of a single particle includes the location and the momentum of that particle. Let us focus on the location of one particle in a box of volume *V*. We have written the locational SMI as:

(24) $$\;H_{max}\left(\mathrm{location}\right)=\log V$$

If we have *N* particles and the locations of all the particles are independent, we can write:

(25) $$\;H_{max}\left(\mathrm{locations}\;\mathrm{of}\;N\;\mathrm{particles}\right)=\overset{N}{\underset{i=1}{\sum }}H_{max}\left(\mathrm{one}\;\mathrm{particle}\right)$$

However, when the particles are indistinguishable, we have the correct Equation (25).

We can define the mutual information corresponding to the correlation between the particles as:

(26)  I(1, 2,…,N)=lnN!

Thus, instead of (25), we will have for SMI for *N* indistinguishable particles, will have:

(27) $$\;H\left(N\;particles\right)=\overset{N}{\underset{i=1}{\sum }}H\left(one\;particle\right)-\ln N!$$

Using the SMI for the location and momentum of one particle in (22), we can write the final result for the SMI of *N* indistinguishable (but non-interacting) particles as:

(28)  H(N indistinguishable particles)=Nlog V(2πmekBTh2)32−logN!

Using the Stirling approximation for logN! (note again that we use the natural logarithm) in the form:

(29)  logN!≈Nlog N−N

We have the final result for the SMI of *N* indistinguishable particles in a box of volume *V*, and temperature *T*:

(30)  H(1,2,…N)=Nlog [VN(2πmkBTh2)32]+52N

By multiplying the SMI of *N* particles in a box of volume *V*, at temperature *T*, by a constant factor ($k_{B}$, if we use the natural log, or $k_{B}\log_{e}2$ if the log is to the base 2), one gets the entropy, the thermodynamic entropy of an ideal gas of simple particles. This equation was derived by Sackur and by Tetrode in 1912, by using the Boltzmann definition of entropy.

One can convert this expression into the entropy function S(E,V,N), by using the relationship between the total kinetic energy of the system, and the total kinetic energy of all the particles:

(31) $$\;E=N\frac{m{\langle v\rangle }^{2}}{2}=\frac{3}{2}Nk_{B}T$$

The explicit entropy function of an ideal gas is:

(32)  S(E,V,N)=NkBln[VN(EN)32]+32kBN[53+ln(4πm3h2)]

We can use this equation as a definition of the entropy of a system characterized by constant energy, volume, and number of particles. Note that when we combine all the terms under the logarithm sign, we must get a dimensionless quantity.

## 3. The Entropy of a System of Interacting Particles

In this section, we show that whenever we “turn on” the intermolecular interactions at constant *T*, *V*, *N*, the entropy of the system will reduce. We will show that whenever there are interactions, there are also correlations and these correlations may be cast in the form of mutual interactions.

We start with the classical canonical partition function (PF) of a system characterized by the variable *T*, *V*, *N*:

(33) $$\;Q\left(T,V,N\right)=\frac{Z_{N}}{N!\Lambda^{3N}}$$

where $\Lambda^{3}$ is the momentum partition function, and $Z_{N}$ is the configurational PF of the system.

(34) $$\;Z_{N}=\int \cdots \int dR^{N}\exp\left[-\beta U_{N}\left(R^{N}\right)\right]$$

The probability density for finding the particles at a specific configuration $R^{N}=R_{1},\cdots ,R_{N}$ is:

(35) $$\;P\left(R^{N}\right)=\frac{\exp\left[-\beta U_{N}\left(R^{N}\right)\right]}{Z_{N}}$$

where $k_{B}$ is the Boltzmann constant and *T* the absolute temperature. In the following, we put $k_{B}=1$ to facilitate the connection between the entropy change and the change in the SMI.

When there are no intermolecular interactions (ideal gas), the configurational PF is $Z_{N}=V^{N}$, and the corresponding partition function is reduced to:

(36) $$\;Q^{ig}\left(T,V,N\right)=\frac{V^{N}}{N!\Lambda^{3N}}$$

We define the change in the Helmholtz energy due to the interactions as:

(37) $$\;\Delta A=A-A^{ig}=-T\ln\;\frac{Q\left(T,V,N\right)}{Q^{ig}\left(T,V,N\right)}=-T\ln\frac{Z_{N}}{V^{N}}$$

This change in Helmholtz energy corresponds to the process of “turning on” the interaction among all the particles at constant (T,V,N).

The corresponding change in the entropy is:

(38) $$\Delta S=-\frac{\partial \Delta A}{\partial T}=\ln\frac{Z_{N}}{V^{N}}+T\frac{1}{Z_{N}}\frac{\partial Z_{N}}{\partial T}\;=\;\ln Z_{N}-N\mathrm{lnV}+\frac{1}{T}\int dR^{N}P\left(R^{N}\right)U_{N}\left(R^{N}\right)$$

We now substitute $U_{N}\left(R^{N}\right)$ from (35) into (38) to obtain:

(39) $$\Delta S=-N\ln\;V-\int P\left(R^{N}\right)\ln P\left(R^{N}\right)dR^{N}$$

Note that the second term on the *rhs* of (39) has the form of a SMI. We can also write the first term on the *rhs* of (39) as SMI of ideal gas.

For ideal gas $U_{N}\left(R^{N}\right)=0$, and:

(40) $$\;P^{ig}\left(R^{N}\right)={\left(1/V\right)}^{N}P\left(R_{1}\right)P\left(R_{2}\right)\cdots \left(R_{N}\right)$$

Hence:

(41) $$\Delta S=\ln P^{ig}\left(R^{N}\right)-\int P\left(R^{N}\right)\ln P\left(R^{N}\right)dR^{N}$$

Since $P^{ig}\left(R^{N}\right)={\left(1/V\right)}^{N}$ is independent of $R^{N}$, we can rewrite $\ln P^{ig}\left(R^{N}\right)$ as:

(42) $$\;\ln P^{ig}\left(R^{N}\right)=\int P^{ig}\left(R^{N}\right)\ln P^{ig}\left(R^{N}\right)dR^{N}$$

Hence, Equation (41) is rewritten as:

(43) $$\Delta S=-\int P\left(R^{N}\right)\ln\left[\frac{P\left(R^{N}\right)}{P^{ig}\left(R^{N}\right)}\right]dR^{N}=-\int P\left(R^{N}\right)\ln\left[\frac{P\left(R^{N}\right)}{\prod_{i=1}^{N}P\left(R_{1}\right)}\right]dR^{N}$$

The last expression on the *rhs* of (43) has the form of mutual information. We define the correlation function among the *N* particles as:

(44) $$\;g\left(1,\;2,\;\cdots ,\;N\right)=\frac{P\left(R^{N}\right)}{\prod_{i=1}^{N}P\left(R_{1}\right)}$$

Using (44) in (45) we get the final form of the entropy change:

(45) $$\;\Delta S=-\int P\left(R^{N}\right)\ln g\left(R^{N}\right)d\left(R^{N}\right)=-I\left(1,\;2,\;\cdots ,\;N\right)$$

Thus, except for the Boltzmann constant and change in the base of the logarithm, ΔS is equal to the negative *mutual information* I(1, 2, ⋯, N), between the locations of the *N* particles. Since the mutual information is always positive (see Ben-Naim, [12]), the change in entropy in (45) is always negative. We can conclude that no matter what the interactions are, whenever we “turn off” the interactions, the entropy of the system will always *increase*.

## 4. Entropy of Non-Ideal Gas

We next derive a particular case of Equation (45), where we know an explicit expression for the correlation functions. 

In the limit of very low density, when only pair interactions are operative, but interactions among more than two particles are rare and can be neglected, we can get a more useful expression for ΔS. We can write the configurational PF as:

(46) $$\;Z_{N}=\int dR^{N}\underset{i<j}{\prod }\exp\left[-\beta U_{ij}\right]$$

where $U_{ij}$ is the pair potential between particles *i* and *j*.

Define the so-called Mayer *f*—*function,* by:

(47) $$\;f_{ij}=\exp\left(-\beta U_{ij}\right)-1$$

and rewrite $Z_{N}$ as:

(48) $$\;Z_{N}=\int dR^{N}\underset{i<j}{\prod }\left(f_{ij}+1\right)=\int dR^{N}\left[1+\underset{i<j}{\sum }f_{ij}+\sum f_{ij}f_{jk}+\cdots \right]$$

Neglecting all terms beyond the first sum, we obtain:

(49) $$\;Z_{N}=V^{N}+\frac{N\left(N-1\right)}{2}\int f_{12}dR^{N}=V^{N}+\frac{N\left(N-1\right)}{2}V^{N-2}\int f_{12}dR_{1}dR_{2}$$

We now identify the second virial coefficient as:

(50) $$\;B_{2}\left(T\right)=\frac{-1}{2V}\int f_{12}dR_{1}dR_{2}$$

and rewrite $Z_{N}$ as:

(51) $$\;Z_{N}=V^{N}-N\left(N-1\right)V^{N-1}B_{2}\left(T\right)=V^{N}\left[1-\frac{N\left(N-1\right)}{V}B_{2}\left(T\right)\right]$$

The corresponding Helmholtz energy change is:

(52) $$\Delta A=A-A^{ig}=-T\ln\;\frac{Z_{N}}{V^{N}}=-T\ln\;\left[1-\frac{N\left(N-1\right)}{2V^{2}}\iint f_{12}\left(R_{1},R_{2}\right)dR_{1}dR_{2}\right]$$

Since we have engaged the low-density limit, we can rewrite (52) as:

(53) $$\Delta A\approx T\frac{N\left(N-1\right)}{2V^{2}}f_{12}\left(R_{1},R_{2}\right)dR_{1}dR_{2}$$

Note that $\frac{N\left(N\;-\;1\right)}{V^{2}}\approx \rho^{2}$, hence we can use the approximation $\ln\left(1-\rho^{2}B\right)\approx -\rho^{2}B$, where *B* is the integral in (52).

In this limit, the entropy change for the process of “turning on” the interactions is:

(54) $$\Delta S\approx -\frac{N\left(N-1\right)}{2V^{2}}\times [\int f_{12}\left(R_{1},R_{2}\right)dR_{1}dR_{2}+T\int \frac{\partial f_{12}}{\partial T}dR_{1}dR_{2}]$$

We now use the following limiting behavior of the pair distribution and the pair correlation function:

(55) $$\;P\left(R_{1},R_{2}\right)=\frac{g\left(R_{1},R_{2}\right)}{V^{2}}$$

(56) $$\;g\left(R_{1},R_{2}\right)=\exp\left[-\beta U_{12}\right]$$

We can rewrite (54) as:

(57) $$\Delta S=-\frac{N\left(N-1\right)}{2}\int P\left(R_{1},R_{2}\right)\ln g\left(R_{1},R_{2}\right)dR_{1}dR_{2}$$

Thus, except for the base of the logarithm, the internal in Equation (57) is the mutual information for a pair of particles. In a system of *N* particles, there are altogether $\frac{N\left(N\;-\;1\right)}{2}$ pairs of particles. Therefore, the entropy change, up to a constant, is the mutual interaction between all the pairs of particles in the system, i.e.,

(58) $$\Delta S=-\frac{N\left(N-1\right)}{2}I\left(R_{1};R_{2}\right)$$

Since the mutual information is always positive, ΔS will be negative.

Thus, we see again that “turning on” the interaction will reduce the entropy of the system.

It should be noted that the correlation function can be either positive (i.e., lng≥0) or negative (i.e., lng≤0), but the average in (57) must always be positive. For more details, see Ben-Naim [15].

## 5. Discussion and Conclusions

We have seen that experimentally the entropy change of vaporization is always positive. Traditionally, this fact is interpreted in terms of relative disorder of the gaseous phase compared with the liquid phase. However, using the informational interpretation of entropy, we can interpret the change in entropy as consisting of two steps.

We define the entropy of vaporization as the difference in entropy for transferring one mole of the substance from the liquid state to the gaseous phase at equilibrium. Thus, we write:

(59) $$\Delta S_{v}=S\left(T,V_{g},N\right)-S\left(T,V_{l},N\right)$$

where *N* is the Avogadro number, *T* is the temperature of the two phases, and $V_{g}$ and $V_{l}$ are the molar volumes of the substance in the two phases, with $V_{g}\gg V_{l}$.

**Figure 3:**
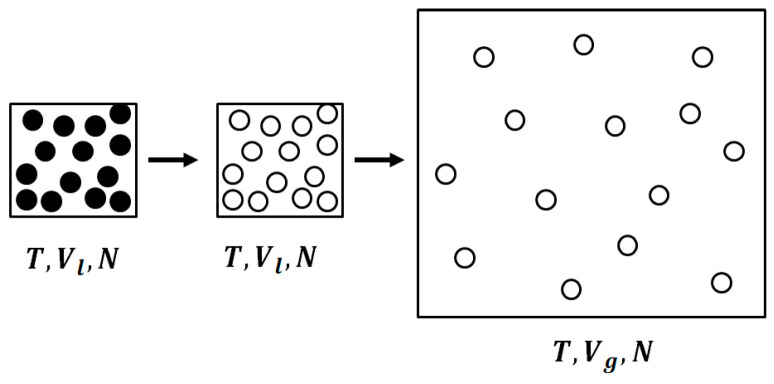
The two-step transition from a liquid to an ideal gas; First, the interactions are “turned off” at constant volume *V_l_*, then there is an expansion to a larger molar volume, *V_g_*.

Our interpretation of the entropy of vaporization consists of two steps, shown schematically in Figure 3. We start with one mole at $\left(T,V_{l},N\right)$ with entropy value $S^{l}=S\left(T,V_{l},N\right)$. We first “turn off” the interactions at constant *T* and *V*. The resulting change in entropy is:

(60) $$\Delta S\left(\mathrm{turning}\;\mathrm{off}\;\mathrm{the}\;\mathrm{interactions}\right)=S^{ig}\left(T,V_{l},N\right)-S^{l}\left(T,V_{l},N\right)\geq 0$$

This change in entropy is due to the correlation among the particles. The second step is to expand the mole of ideal gas from the volume $V_{l}$ to the volume $V_{g}$, i.e.,

(61) $$\Delta S\left(\mathrm{expansion}\right)=S^{ig}\left(T,V_{g},N\right)-S^{ig}\left(T,V_{l},N\right)\geq 0$$

Thus, the entropy of vaporization consists of two contributions: one, due to mutual information; and the second, due to the expansion of the ideal gas from $V_{l}$ to $V_{g}$.

Both of these contributions are positive, and both are interpreted in terms of increasing the value of the locational SMI (the momentum SMI is constant for this process). We note here that water has a larger entropy of vaporization compared to other “normal” liquids. Again, this fact is traditionally interpreted as due to the structure of water. Our interpretation of the entropy of vaporization of water is based on the strong intermolecular interactions among the water molecules (hydrogen bond), which lead to stronger correlations among the water molecules. This interpretation applies to any hydrogen-bonded liquid such as methanol and ethanol, as can be seen from Table 1. For these liquids, one cannot invoke the “structure” to explain the large entropy of vaporization. The informational interpretation, however, is the same; namely, strong hydrogen bonds lead to strong correlations, hence, mutual information.

## Figures and Tables

**Table 1:** Entropies of vaporization of liquids at their normal boiling point.

	$\Delta S_{v}/J\;mol^{-1\;}K^{-1}$
Benzene	+87.2
Carbon disulfide	+83.7
Carbon tetrachloride	+85.8
Cyclohexane	+85.1
Dimethyl ether	+86.0
Methane	+73.2
Methanol	+104.1
Ethanol	+110.0
Water	+109.1
